# Book Reviews

**Published:** 1869-07

**Authors:** 


### BIBLIOGRAPHICAL NOTICES.

---

*Atlas Zur Pathologie Der Zahn, Bearbeitet Von Weil. Prof. Dr. M. Heider und Prof. Dr. C. Wedl.*—Die Zeichnunger Sammtlich Nach Der Natur Aufgenommen Von Dr. C. Heitzmann. Dritte Lieferung. Leipzig: Verlag Von Arthur Felix.

Atlas to the Pathology of the Teeth. Arranged and explained by the late Prof. Dr. M. Heider and Prof. C. Wedl. All the drawings are taken from nature by Dr. C. Heitzmann.

Part I and II of this interesting work have already been noticed in the May No. of the *Journal*, and we have now before us another number. Part III contains four fine lithographic plates, representing thirty-two pathological specimens of enamel, dentine and cementum accompanied with explanatory notes in English as well as in German. One more number completes the volume which will prove a valuable addition to the library of the dental practitioner.

---

*Proceedings of the State Medical Society of Michigan for* 1867-68.—These "Proceedings" contain many interesting reports on various subjects connected with the practice of medicine and surgery, and may be read with profit.

---

*Half Yearly Compendium of Medical Science.*—S. W. Butler, M. D., Philadelphia. The great value of this work is so universally acknowledged by the members of the medical profession that we need not dwell upon its merits. The dental practitioner who wishes to keep himself posted in medical literature will find no better instructor than the "Half Yearly Compendium," The contents of each number consist of Anatomy and Physiology, Physics, Botany, Chemistry, and Toxicology, Materia Medica and Therapeutics, General Medicine, Clinical Medicine, Obstetrics, and Diseases of Women and Children, and Surgery.

